# *Lactobacillus reuteri* Surface Mucus Adhesins Upregulate Inflammatory Responses Through Interactions With Innate C-Type Lectin Receptors

**DOI:** 10.3389/fmicb.2017.00321

**Published:** 2017-03-07

**Authors:** Krisztián P. Bene, Devon W. Kavanaugh, Charlotte Leclaire, Allan P. Gunning, Donald A. MacKenzie, Alexandra Wittmann, Ian D. Young, Norihito Kawasaki, Eva Rajnavolgyi, Nathalie Juge

**Affiliations:** ^1^Department of Immunology, Faculty of Medicine, University of DebrecenDebrecen, Hungary; ^2^The Gut Health and Food Safety Programme, Institute of Food ResearchNorwich, UK; ^3^Food and Health Programme, Institute of Food ResearchNorwich, UK

**Keywords:** *L. reuteri*, probiotic, mucus adhesins, dendritic cells, C-type lectins, DC-SIGN, Dectin-2, T-lymphocytes

## Abstract

The vertebrate gut symbiont *Lactobacillus reuteri* exhibits strain-specific adhesion and health-promoting properties. Here, we investigated the role of the mucus adhesins, CmbA and MUB, upon interaction of *L. reuteri* ATCC PTA 6475 and ATCC 53608 strains with human monocyte-derived dendritic cells (moDCs). We showed that mucus adhesins increased the capacity of *L. reuteri* strains to interact with moDCs and promoted phagocytosis. Our data also indicated that mucus adhesins mediate anti- and pro-inflammatory effects by the induction of interleukin-10 (IL-10), tumor necrosis factor alpha (TNF-α), IL-1β, IL-6, and IL-12 cytokines. *L. reuteri* ATCC PTA 6475 and ATCC 53608 were exclusively able to induce moDC-mediated Th1 and Th17 immune responses. We further showed that purified MUB activates moDCs and induces Th1 polarized immune responses associated with increased IFNγ production. MUB appeared to mediate these effects *via* binding to C-type lectin receptors (CLRs), as shown using cell reporter assays. Blocking moDCs with antibodies against DC-specific intercellular adhesion molecule 3-grabbing non-integrin (DC-SIGN) or Dectin-2 did not affect the uptake of the MUB-expressing strain, but reduced the production of TNF-α and IL-6 by moDCs significantly, in line with the Th1 polarizing capacity of moDCs. The direct interaction between MUB and CLRs was further confirmed by atomic force spectroscopy. Taken together these data suggest that mucus adhesins expressed at the cell surface of *L. reuteri* strains may exert immunoregulatory effects in the gut through modulating the Th1-promoting capacity of DCs upon interaction with C-type lectins.

## Introduction

The gastrointestinal (GI) tract harbors a vast and diverse community of commensal bacteria (microbiota) which has co-evolved with the host (Walter and Ley, 2011). The gut microbiota provides a range of benefits to the host including the digestion of complex carbohydrates, production of metabolites, detoxification, protection against pathogens, and the development and regulation of the immune system (Sun and Chang, 2014). The intestinal immune system negotiates a delicate balance between immunogenicity against invading pathogens and tolerance to the commensal microbiota (Mann and Li, 2014). *Lactobacillus reuteri* is a common inhabitant of the GI tract of vertebrates and displays remarkable host adaptation. *L. reuteri* has diversified into separate phylogenetic clades reflecting host origin (Oh et al., 2010) with genomic differences reflecting the niche characteristics of the host GI tract (Frese et al., 2011). We previously reported that the adhesion of *L. reuteri* strains to mucus is strain-specific, correlating with the presence of host-clade mucus-binding proteins (MacKenzie et al., 2010; Etzold et al., 2014b). *L. reuteri* adhesins include mucus-binding proteins, MUB (Roos and Jonsson, 2002; MacKenzie et al., 2009, 2010; Etzold et al., 2014a) and CmbA (Jensen et al., 2014; Etzold et al., 2014b), and serine-rich-repeat (SRR) proteins (Frese et al., 2013; Wegmann et al., 2015). *L. reuteri* exhibits strain-specific beneficial properties relevant to human health, including exclusion and inhibition of the growth of intestinal pathogens, maintenance of the gut barrier integrity, and modulation of the host immune system at both local and systemic levels (as reviewed in Walter et al., 2011).

Dendritic cells (DCs) are pivotal in the initiation of adaptive immune responses and can directly contact and internalize intestinal bacteria (Rescigno et al., 2001). Further, DCs can undergo tissue conditioning by intestinal epithelial cells that control the DC inflammatory potential (Iliev et al., 2009). Accordingly, the intestinal milieu represents a unique environment conditioned by all-*trans* retinoic-acid (ATRA), where metabolite production is increased by peroxisome proliferator-activated receptor gamma (PPARγ) in both CD1a^-^ CD1d^+^ human monocyte-derived DCs (moDCs) *in vitro* (Szatmari et al., 2006; Gogolak et al., 2007) and in human intestinal DCs (Gyöngyösi et al., 2013). T-lymphocytes primed by DCs with monocyte precursors play an important role in the maintenance of self-tolerance against gut commensal bacteria (Persson et al., 2013). DCs use pattern recognition receptors (PRRs), such as Toll-like receptors (TLRs) or C-type lectin receptors (CLRs) to sense various microbe-associated molecular patterns (MAMPs). In the gut, DCs are able to distinguish between different members of the microbiota (Diebold, 2009; Feng et al., 2012) and drive the activation and differentiation of naive T-lymphocytes into either effector (Th1, Th17) or regulatory T cells (Treg) (Geijtenbeek and Gringhuis, 2009; Rescigno, 2014). In addition, the nature of T-lymphocyte polarizing signals is largely determined by the type of microbial products, inflammatory signals, or both encountered in peripheral tissues during the immature phase (Geijtenbeek and Gringhuis, 2009; Hooper and Macpherson, 2010; Rescigno, 2014).

*L. reuteri* has been shown to have immunomodulatory properties and promote mucosal tolerance in the vertebrate GI tract. Specific probiotic strains of *L. reuteri* were recently shown to suppress intestinal inflammation in a trinitrobenzene sulfonic-acid (TNBS)-induced mouse colitis model *via* down-regulation of gene expression of the mucosal cytokine IL-6 and IL-1β in the colon (Gao et al., 2015). *L. reuteri* 100-23 stimulated the development of an increased number of regulatory T cells in mice (Livingston et al., 2010). Immunomodulation was also reported in piglets following oral administration of *L*. *reuteri* I5007, resulting in an increased level of TGF-β and a decrease in IFNγ gene expression in the mesenteric lymph nodes (Hou et al., 2015). In humans, *L. reuteri* ATCC 55730 was shown to temporarily colonize the stomach and the small intestine of healthy subjects and thus increase CD4^+^ helper T-lymphocyte numbers in the ileum (Valeur et al., 2004). However, the modulation of cytokine production by *L. reuteri* appears to be strain-dependent, as demonstrated *in vitro*. For example, anti-inflammatory *L. reuteri* strains ATCC PTA 6475 and ATCC PTA 5289, but not the immuno-stimulatory *L. reuteri* strains ATCC 55730 and CF48–3A, suppressed TNF-α production induced by bacterial lipopolysaccharide (LPS)-activated monocytic cells (Lin et al., 2008; Jones and Versalovic, 2009). Moreover, a recent study showed that *L. reuteri* strains from human-associated clades differed in their ability to modulate human cytokine production (TNF-α, monocyte chemoattractant protein-1 (MCP-1), IL-1β, IL-5, IL-7, IL-12, and IL-13) by myeloid cells (Spinler et al., 2014). The bacterial molecule(s) responsible for down-regulating TNF-α in antigen presenting cells have not been identified to date but appear to be strain-specific (Lin et al., 2008; Jones and Versalovic, 2009).

Recently, it was suggested that cell-surface proteins may play a role in regulating the immunomodulatory properties of lactobacilli (Lebeer et al., 2008; Meijerink et al., 2010; Remus et al., 2013; Call et al., 2015; Jensen et al., 2015). However, the detailed molecular mechanisms by which *L. reuteri* may interact with DCs to modulate immune responses and promote mucosal homeostasis are not well understood. The aim of this work was to investigate the role of *L. reuteri* strain-specific mucus adhesins on the modulation of pro- and anti-inflammatory cytokine production by human moDCs and the participation of CLRs in this process.

## Materials and Methods

### Bacterial Strains and Reagents

*L. reuteri* ATCC PTA 6475 and *L. reuteri* ATCC PTA 6475 lar_0958 KO mutant (6475-KO) (Etzold et al., 2014b), and *L. reuteri* ATCC 53608 and 1063N isolates (MacKenzie et al., 2010) were used in this study. The anti-Mub antibody is described in MacKenzie et al. (2010). SIGN-R1 was purchased from Sino Biological (Beijing, China), DC-SIGN from Caltag-Medsystems Ltd (Buckingham, UK), and Dectin-1 and Dectin-2 from R&D Systems (Abingdon, UK). The anti-human Dectin-2 (HDECT2) IgG was from Invitrogen (Toulouse, France) and the DC-SIGN/CD209 mAb (clone 120507) from Abcam, (Cambridge, UK).

### Human moDC Cultures

Leukocyte-enriched buffy coats were obtained from healthy blood donors at the Regional Blood Center of the Hungarian National Blood Transfusion Service (Debrecen, Hungary) in accordance with the written approval of the Director of the National Blood Transfusion Service of the University of Debrecen, Faculty of Medicine (Hungary). Peripheral blood mononuclear cells (PBMCs) were separated by a standard density gradient centrifugation with Ficoll-Paque Plus (Amersham Biosciences, Uppsala, Sweden). Monocytes were purified from PBMCs by positive selection using immunomagnetic cell separation using anti-CD14 microbeads, according to the manufacturer’s instruction (Miltenyi Biotec, Bergisch Gladbach, Germany). After separation on a VarioMACS magnet, 96–99% of the cells were shown to be CD14^+^ monocytes, as measured by flow cytometry. Isolated monocytes were cultured in 12-well tissue culture plates at a density of 5.0 × 10^5^ cells/ml in Gibco’s serum-free AIM-V medium (Invitrogen, Carlsbad, CA, USA), supplemented with 80 ng/ml granulocyte-monocyte stimulating factor (GM-CSF) (Gentaur Molecular Products, Brussels, Belgium) and 100 ng/ml interleukin-4 (IL-4) (PeproTech EC, London, UK). The cells were differentiated in the presence or absence of 1 nM ATRA (Sigma–Aldrich, Schnelldorf, Germany) at 37°C atmosphere containing 5% CO_2_.

### Phagocytosis Assay

Live bacterial cells were centrifuged at 1000 × *g* for 5 min and washed three times in 25 ml phosphate buffered saline (PBS). Suspensions of bacterial cells were heat inactivated by heating at 65°C for 45 min and re-suspended in 0.25 M carbonate-bicarbonate buffer (pH 9.0). The bacterial cell suspensions (900 μl) were stained with 100 μl fluorescein-isothiocyanate (FITC) dissolved at 5 mg/ml in dimethyl-sulfoxide (DMSO) and were rotated overnight at 4°C in dark. FITC-labeled bacteria were washed three times with cold PBS and were co-incubated for 3 h with moDCs at 37°C or 4°C at a moDC:bacteria ratio of 1:20. The moDCs positive for FITC-labeled bacteria were analyzed by flow cytometry.

### Growth of Bacteria for moDC Activation

*L. reuteri* strains were grown in Difco^TM^ de Man, Rogosa and Sharpe (MRS) broth medium for 18 h stationary at 37°C (MRS, BD BioSciences, Franklin Lakes, NJ, USA). Bacterial suspensions were washed with 25 ml sterile PBS buffer three times and OD_600__nm_ was measured by spectrophotometry and converted to cell/ml following OD_600__nm_ × 2.5 × 10^8^ CFU/ml (Regulski et al., 2012). Human moDC cultures were activated with LPS as control (250 ng/ml ultrapure LPS, InvivoGen, 3950 Sorrento Valley Blvd, San Diego, CA, USA) or with live *L. reuteri* strains at a non-toxic ratio of 0.4:1 bacteria:moDCs and were co-cultured for another 24 or 1.5 h.

### Flow Cytometry Analysis

Phenotyping of resting and activated moDCs was performed by flow cytometry using anti-HLA-DR-PE (BD Biosciences), anti-CD80-FITC, anti-CD83-FITC, anti-CD86-PE (R&D Systems, Minneapolis, MN, USA) and isotype-matched control antibodies. To block non-specific antibody binding, heat-inactivated mouse serum was used. Fluorescence intensities were measured by FACS Calibur (BD Biosciences). The data were analyzed by the FlowJo software version 5.7.1 (Tree Star, Ashland, OR, USA).

### Measurement of Cytokine Concentrations

Culture supernatants of moDCs were harvested 24 h after moDC activation, and the concentration of TNF-α, IL-1β, IL-6, IL-10, IL-12(p70) and IL-23(p19) cytokines was measured using OptEIA kits (BD Biosciences, Franklin Lakes, NJ, USA) following the manufacturer’s instructions.

### Measurement of the Polarization of IFNγ and IL-17-Producing T-Lymphocytes

Activated moDCs were washed and co-cultured with peripheral blood lymphocytes (PBLs) for 4 days in AIM-V medium at moDC:T-cell ratios of 1:20. IFNγ and IL-17 secretion was analyzed using the avidin-horseradish peroxidase based enzyme-linked ImmunoSpot (ELISPOT) system (NatuTec GmbH, Herriotstrasse 1, 60528 Frankfurt am Main. Germany). Lymphocytes activated by phytohaemagglutinin or concanavalin A were used as positive controls, the co-cultures containing resting moDCs and T-cells, or T-cells alone served as negative controls. To detect IL-17 secretion, the plates were coated with 0.5 μg/ml murine anti-human CD3 antibody (BD Biosciences). The plates were analyzed by using the ImmunoScan plate reader (Cell Technology Limited, Shaker Heights, OH, USA).

### MUB Purification

*L. reuteri* ATCC 53608 was inoculated from −80°C glycerol stocks into the semi-defined substrate medium, LDMII (Kotarski and Savage, 1979) under anaerobic conditions for 16 h at 37°C, followed by sub-culture at 0.1% (v/v) for 24 h at 37°C to stationary phase. Cells were removed by centrifugation at 7,500 × *g* for 15 min at 4°C. Spent culture medium was further purified by vacuum-filtration through 0.45 μm and 0.22 μm filter disks (Merck Millipore, Nottingham, UK), sequentially, and concentrated by tangential flow filtration using Vivaflow 200 cassettes (100,000 Da molecular weight cut-off; Sartorius Stedim, Surrey, UK). The spent culture was further purified by gel filtration chromatography using a Superose 6 GL 10/300 column (GE Healthcare, Little Chalfont, UK) with PBS as eluent. To fractionate MUB from co-purifying glycolipid, CHAPS (3-[(3-cholamidopropyl)dimethylammonio]-1-propanesulfonate; Sigma Aldrich, Darmstadt, Germany) was added to 0.5% (w/v) to both the sample and eluent during gel filtration. The MUB protein (predicted size of 353 kDa) was eluted in the void volume of the column with and without the addition of CHAPS and its purity was confirmed by SDS-PAGE. Before use, MUB was dialyzed extensively against PBS using Vivaspin 6 spin filters (100 kDa; Sartorius Stedim Ltd, Surrey, UK) to remove CHAPS.

### Endotoxin Removal

The MUB protein solution was further purified according to the protocol of Zhang et al. (2005). Briefly, Lipid Removal Adsorbent (2% w/v; Sigma Aldrich, Darmstadt, Germany) was added to the MUB solution and was incubated under gentle rotation for 2 h at 4°C. The mixture was then centrifuged at 5,000 × *g* for 10 min at 4°C and the supernatant collected and stored at 4°C until use.

### Cell Reporter Assay

BWZ.36 cells expressing murine C-type lectins receptors (CLRs), mouse Dectin-1 (mDectin-1), and SIGN-R1 were established as described previously for mouse Dectin-2 (mDectin-2) (Wittmann et al., 2016). Briefly, the extracellular domain of mDectin-1 (Ser74 through Leu244) and SIGN-R1 (Ser76 through Gly324) were cloned separately into the retrovirus vector pMXs-IRES-EGFP-Ly49A-CD3ζ encoding transmembrane region of the mouse Ly49A and the cytoplasmic domain of the mouse CD3ζ (Wittmann et al., 2016). The resulting pMXs vectors encoding mDectin-1 and SIGN-R1, respectively, were used for the retrovirus transduction using Plat-E cells (Wittmann et al., 2016). In order to introduce two amino acid mutations into the carbohydrate-binding domain of SIGN-R1 (E285Q and D287N), a DNA fragment encoding the extracellular domain of SIGN-R1 with the two missense mutations was synthesized (Genscript) and used for cloning as above.

To assess the binding of MUB or lipid component to CLRs, ligands were adsorbed onto 96-well microplates (Nunc) overnight at 4°C. The MUB protein was added at 0.1 mg/ml in PBS, and the lipid fraction co-purified with the MUB protein was diluted with PBS at a ratio of 1:1 before adsorption to the plate. Scleroglucan (1 μg/ml) (ELICITYL), α-mannan (2 μg/ml) (Sigma–Aldrich), and Hafnia-LPS (1 μg/ml) (a gift from Dr. Ewa Katzenellenbogen, Ludwik Hirszfeld Institute of Immunology and Experimental Therapy, Wroclaw, Poland) were used as specific ligands for mDectin1, mDectin-2 and SIGN-R1, respectively. Wells were washed twice with PBS, and reporter cells (1 × 10^6^ cells/ml) and were added to each well and incubated for 18 h at 37°C, 5% CO_2_. Following incubation, the microwell plates were centrifuged at 510 × *g* for 3 min and the supernatant was discarded. β-galactosidase activity (encoded by the *lacZ* reporter gene) was determined by the addition of 200 μL of 150 mM chlorophenol red-β-D-galactopyrasonide (CPRG; Roche) diluted in a CPRG assay reaction buffer (PBS supplemented with 0.125% Triton X-100 and 100 mM 2-mercaptoethanol, Lonza) to each well. The plate was incubated for 45 min at 37°C, 5% CO_2_ prior to measurement of color development (A570/630 nm) with a Bio-Rad Benchmark Plus microtiter plate reader.

### Adhesion Measurements by Force Spectroscopy

The interactions between MUB and CRLs were examined by covalently attaching the MUB molecules to atomic force microscopy (AFM) tips and DC-SIGN or SIGN-R1 or Dectin-2 to the glass slides to enable binding interactions to be measured in a specific manner (Hinterdorfer et al., 2002). Silicon nitride AFM tips (PNP-TR, Nanoworld AG, Neuchâtel, Switzerland) and freshly cleaned glass slides were functionalized using a four step procedure (carried out at 21°C): the first step involved incubation in a 2% solution of 3-mercaptopropyltrimethoxy silane (MTS, Sigma–Aldrich, Poole, Dorset, UK) in toluene (dried over a 4Å molecular sieve) for 2 h, followed by washing with toluene and then chloroform. In the second step, the silanised tips were incubated for 1 h in a 1 mg/ml solution of a heterobifunctional linker: MAL-PEG-SCM, 2 kD (Creative PEGWorks, Chapel Hill, NC, USA) in chloroform. The silanised glass slides were incubated in 5 mM *N*-γ-maleimidobutyryl-oxysuccinimide ester (GMBS) in ethanol (Thermo Fisher Scientific, Waltham, MA, USA). The tips and slides were rinsed with chloroform/ethanol, respectively, and then dried with argon. The third step involved covalent attachment of the molecules investigated in this study (i.e., CLRs and MUB) by incubation of the tips/slides in 1 mg/ml solutions of the proteins in PBS at pH 7.4 for 1 h at 21°C, followed by a PBS washing step. The fourth step involved incubation of the functionalized cantilevers/slides in a 10 mg/ml solution of glycine in PBS to ‘amine’-cap any unreacted succinimide groups, followed by washing in PBS.

Binding measurements were carried out in PBS using a MFP-3D BIO AFM (Asylum Research Inc., Santa Barbara, CA, USA). The experimental data were captured in ‘force-volume’ (FV) mode at a rate of 2 μm/s in the Z direction and at a scan rate of 1 Hz and a maximum load force of 300 pN (pixel density of 32 × 32). The spring constant, k, of the cantilevers was determined by fitting the thermal noise spectra (Hutter and Bechhoefer, 1993), yielding typical values in the range 0.03–0.06 N/m. Adhesion in the force spectra was quantified using a bespoke Excel macro (Gunning et al., 2008) which fits a straight line to the baseline of the retract portion of the force-distance data. In order to explore the specificity of the binding interactions, the force measurements were repeated after addition of 0.05 mg/ml anti-Mub antibody (MacKenzie et al., 2010) to the liquid cell.

### *In vitro* Detection of CLR-Mediated Inflammatory Responses

To assess the role of CLRs in the induction of moDC-mediated inflammatory cytokine release, endotoxin-free purified MUB was coated to sterile high protein-affinity OptEIA ELISA 96-well plates (BD Biosciences) at 38.5 μg/ml concentration followed by overnight incubation at 4°C. After repeated washing steps with sterile PBS, the cells were co-incubated with moDCs in the presence or absence of 5 μg/ml anti-Dectin-2 monoclonal antibody (clone Q7-4B5 InvivoGen) or anti-DC-SIGN (120507 Abcam, Cambridge, UK) antibody for 1 h on ice. After the washing steps with 5 ml fresh AIM-V medium 2 × 10^5^ moDCs were cultured in microwell plastic plates for 24 h coated or uncoated with MUB. The concentration of TNF-α and IL-6 cytokines was measured in the culture supernatants using OptEIA kits. In another set of experiments, moDCs were first co-incubated with blocking antibodies specific for Dectin-2 or DC-SIGN and following a washing step with fresh AIM-V medium, the cells were co-cultured with *L. reuteri* ATCC 53608 or 1063N strains for 1.5 h at 37°C at a moDC:bacteria ratio of 1:4. The supernatant was removed by centrifugation at 1000 rpm for 5 min and the cells washed repeatedly with 5 ml PBS at 4°C (five times) followed by centrifugation at 1000 rpm. Finally, the moDCs were co-cultured with autologous T-lymphocytes in AIMV medium for 4 days at a moDC:T-cell ratio of 1:20. The secretion of IFNγ was analyzed by ELISPOT assay. T-lymphocytes co-cultured with either resting moDCs, bacteria or culture media served as negative controls.

### Statistical Analysis

Student’s unpaired two-tailed *t*-test or ANOVA followed by Bonferroni’s multiple comparison tests were used as indicated in the relevant experiments. In case of significantly different variances (*p* < 0.05) between the sample sets the Welch’s correction was applied in the *t*-test. The results were expressed as mean + SD. All analyses were performed by using the GraphPad Prism software, version 6.0. Differences were considered to be statistically significant at *P* < 0.05. Significance was indicated as ^∗^*P* < 0.05; ^∗∗^*P* < 0.01; ^∗∗∗^*P* < 0.005; ^∗∗∗∗^*P* < 0.0001.

## Results

### Mucus Adhesins Facilitate the Phagocytosis of *L. reuteri* Strains by moDCs

The *L. reuteri* strains ATCC PTA 6475 and ATCC 53608 (1063) express mucus-binding proteins CmbA (Jensen et al., 2014; Etzold et al., 2014b) and MUB (Etzold et al., 2014a; MacKenzie et al., 2010), respectively on their cell surface. It is well established that the phagocytic process of bacteria can modulate the outcome of immune responses. To assess the impact of mucus adhesins on the internalization of *L. reuteri* strains by moDCs, we used *L. reuteri* mutant strains, *L. reuteri* 6475-KO (Jensen et al., 2014; Etzold et al., 2014b) and 1063N (MacKenzie et al., 2010) deficient for CmbA and MUB, respectively, in comparison to the wild-type strains. In these experiments, the moDCs isolated from PBMCs of healthy blood donors were cultured in serum-free medium in the presence or absence of ATRA.

**FIGURE 1:**
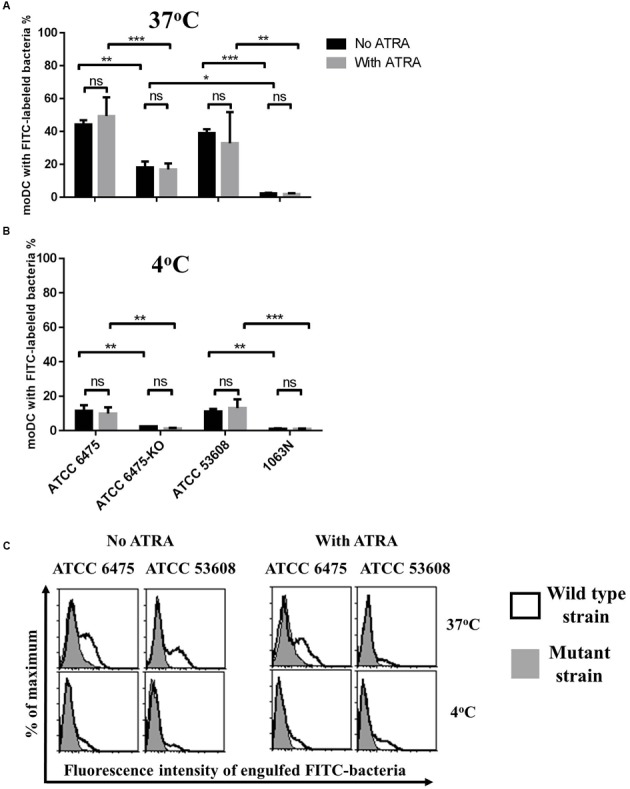
**The phagocytic capacity of moDCs co-cultured with *L. reuteri* is strain-dependent.** Human moDCs were differentiated in the presence of GM-CSF, IL-4 and with or without 1 nM ATRA for 2 days. On day 2, moDCs were co-cultured with heat-inactivated bacteria at 37°C **(A)** or at 4°C **(B)** for 1.5 h at a moDC:bacteria ratio of 1:20. Bacterial uptake was measured by flow cytometry. The number of moDC carrying phagocytosed FITC-labeled bacteria was calculated from 3 independent experiments + SD. Histogram overlays are shown for one independent experiment **(C)**. Statistical differences were analyzed by ANOVA, with significance defined as ^∗^*P* < 0.05, ^∗∗^*P* < 0.01, ^∗∗∗^*P* < 0.001, and ^∗∗∗∗^*P* < 0.0001.

When the FITC-labeled strains were incubated with moDCs at 37°C for 1.5 h, the *L. reuteri* 1063N MUB-mutant was internalized 10-fold less efficiently than its wild-type counterpart ATCC 53608, and the engulfment of ATCC PTA 6475 was twofold more efficient than that of the 6475-KO CmbA mutant strain (**Figures 1A,C**). Moreover, there were significant differences (*p* = 0.0142) between mutant strains with a 10-fold decreased level of internalized 1063N mutant strain as compared to the 6475-KO strain. At 4°C, the phagocytic process was significantly inhibited but the ratio of adherent bacteria was higher in the wild-type strains as compared to the mutants (**Figure 1B**). The presence of ATRA inhibited the cell surface expression of CD1a, resulting in CD1a^−^ CD1d^+^ moDCs (data not shown) but no difference could be detected in the *L. reuteri* phagocytic capacity of the cells as compared to moDCs differentiated in the absence of ATRA. Taken together, these results demonstrate that the cell-surface expressed mucus adhesins have the potential to promote the internalization process by moDCs.

### Mucus Adhesins Modulate moDC-Mediated Immune Responses to *L. reuteri* Strains

**FIGURE 2:**
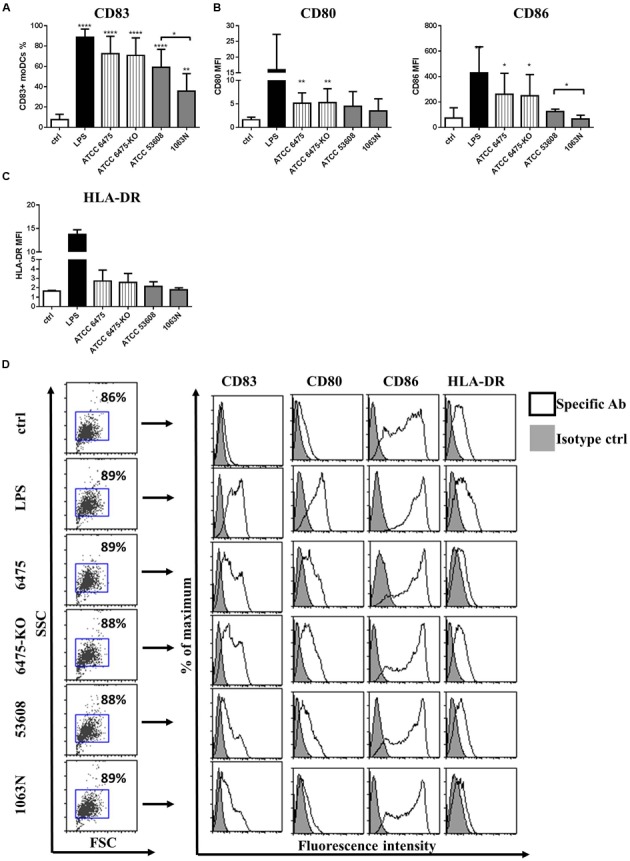
***L. reuteri* strains modulate the expression of cell surface activation markers in moDC.** MoDCs were co-incubated with live *L. reuteri* strains, or LPS as a control, for 24 h. The expression of moDC-associated activation marker CD83 **(A)**, co-stimulatory molecules CD80 and CD86 **(B)** and MHC class II-protein, HLA-DR **(C)** was measured by flow cytometry. Mean values were calculated from 5 to 7 independent experiments ± SD. Histogram overlays are shown for one independent experiment **(D)**. Statistical differences were analyzed by Student’s *t*-test, with significance defined as ^∗^*P* < 0.05, ^∗∗^*p* < 0.01, ^∗∗∗^*P* < 0.001, and ^∗∗∗∗^*P* < 0.0001.

The moDC activating potential of *L. reuteri* ATCC PTA 6475 and ATCC 53608, as well as their mutant strains, was further analyzed by measuring the cell surface expression of CD83, the co-stimulatory molecules CD80 and CD86, and the major histocompatibility complex (MHC) class II-protein, HLA-DR, by flow cytometry. We showed that the cell surface expression of CD83 was induced by all *L. reuteri* strains, however, in the presence of the 1063N mutant, CD83 expression was reduced significantly, suggesting a potential role of MUB in moDC activation (**Figures 2A,D**). Co-culturing moDCs with *L. reuteri* strains for 24 h also induced cell surface expression of the co-stimulatory molecules CD80 and CD86 (**Figure 2B**) and HLA-DR (**Figure 2C**). However, the co-stimulatory potential was exclusively reduced by 1063N.

**FIGURE 3:**
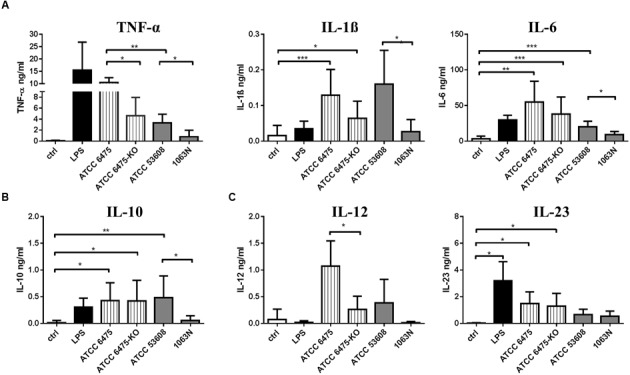
**The secretion of inflammatory and regulatory cytokines is modified in a *L. reuteri* strain-dependent manner in moDCs.** MoDCs were co-incubated with *L. reuteri* strains, or with LPS as a control, for 24 h. The concentration of TNFα, IL-1β, IL-6, **(A)** IL-10 **(B)**, IL-12, IL-23 **(C)** cytokines was measured by ELISA in 5 independent experiments. Mean values ± SD are shown. Statistical differences were analyzed by Student’s *t*-test, with significance defined as ^∗^*P* < 0.05, ^∗∗^*P* < 0.01, ^∗∗∗^*P* < 0.001, and ^∗∗∗∗^*P* < 0.0001.

The level of moDC-derived pro-inflammatory cytokines (TNF-α, IL-1β, IL-6) and that of the T-lymphocyte polarizing cytokines (IL-10, IL-12, IL-23) was monitored upon incubation with wild-type and mutant *L. reuteri* strains. *L. reuteri* ATCC PTA 6475 induced TNF-α secretion more efficiently than ATCC 53608. The 6475-KO and 1063N mutants induced lower levels of TNF-α production than their wild-type counterparts (**Figure 3A**). The *L. reuteri* 1063N mutant was further associated with lower IL-1β, IL-6 and anti-inflammatory IL-10 production as compared to the wild-type ATCC 53608 strain (**Figure 3B**). Interestingly, both *L. reuteri* wild-type ATCC PTA 6475 and mutant 6475-KO induced the secretion of immunoregulatory IL-10 in moDCs to a similar extent whereas the production of the inflammatory cytokines (TNF-α, IL-1β, IL-6) was reduced in presence of 6475-KO. In addition, all *L. reuteri* strains tested in these experiments provoked potent IL-12 and IL-23 cytokine responses as compared to the LPS control (**Figure 3C**). These results demonstrated that mucus adhesins expressed by *L. reuteri* strains have the potential to enhance pro-inflammatory cytokine production in moDCs.

**FIGURE 4:**
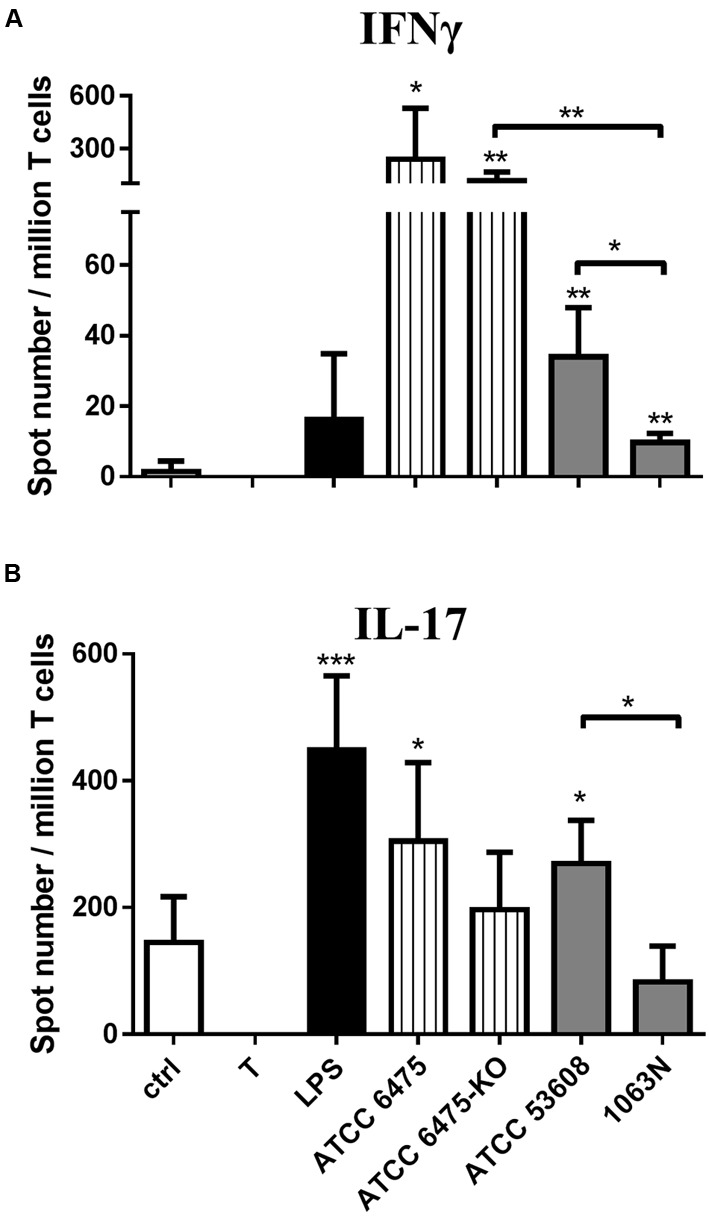
***L. reuteri* mucus adhesins modulate moDC-mediated T-lymphocyte polarization.** The T-cell activating and polarizing capacity of moDCs was monitored by activating moDCs with *L. reuteri* strains or LPS followed by co-culture with T-cells. T corresponds to T-cells cultured without dendritic cells as negative control. The mean values of spot numbers were calculated from five independent experiments + SD. The number of cytokine-producing *T*-lymphocytes induced by LPS or moDCs exposed to *L. reuteri* strains was measured by ELISPOT assays for IFNγ **(A)** and IL-17 **(B)**. Statistical differences were analyzed by Student’s *t*-test, with significance defined as ^∗^*P* < 0.05, ^∗∗^*P* < 0.01, ^∗∗∗^*P* < 0.001, and ^∗∗∗∗^*P* < 0.0001.

Next we addressed the question whether moDC-mediated T-cell responses targeting *L. reuteri* mucus adhesin-expressing strains were able to orchestrate T-lymphocyte polarization. In this context, moDCs were first exposed to *L. reuteri* strains or to LPS used as a control, followed by co-culturing the cells with autologous T-lymphocytes for 4 days. The secretion of IFNγ and IL-17 cytokines was monitored at a single cell level by using the ELISPOT assay with IFNγ or IL-17 specific monoclonal antibody-coated plates. MoDCs activated by the ATCC PTA 6475 or ATCC 53608 wild-type strains induced IFNγ secretion by T-lymphocytes (**Figure 4A**) and resulted in increased IL-17 production (**Figure 4B**), as compared to the immature moDC:T-cell co-cultures. In contrast, the *L. reuteri* 6475-KO and 1063N mutant strains induced IFNγ producing Th1 cell activation, but were unable to trigger IL-17 polarized immune responses. Taken together, these results demonstrate that mucus adhesins are able to up-regulate immune responses against *L. reuteri* strains in moDCs.

### Purified Mucus-Binding Protein (MUB) Triggers CD83 Expression and Provokes Th1 Polarized Immune Responses in moDCs

**FIGURE 5:**
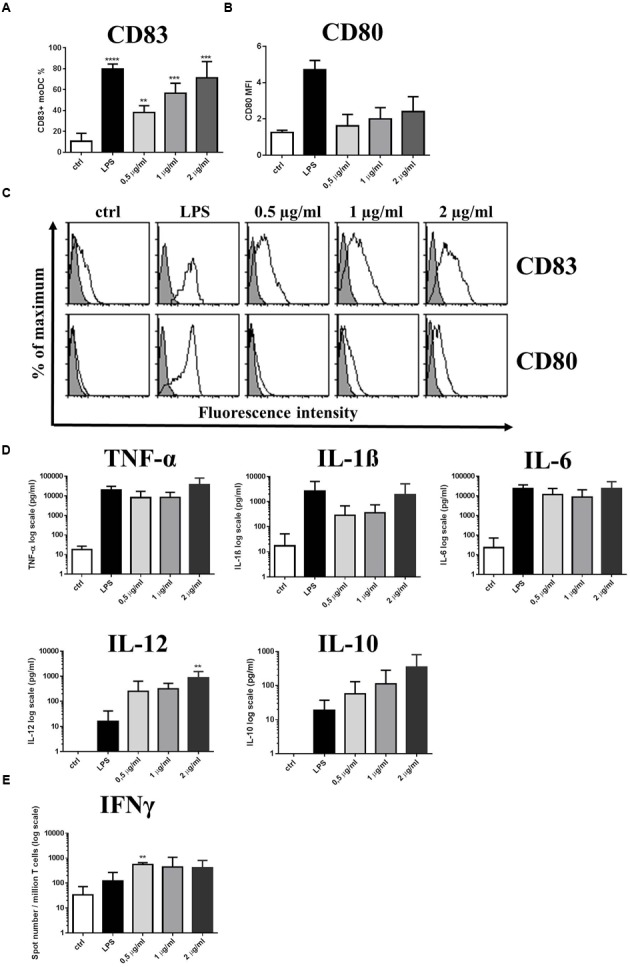
**Purified MUB protein triggers CD83 expression and induces Th1 polarized immune responses.** Increasing concentrations of purified MUB was used to activate 2-day moDC cultures. LPS was used as a positive control. Cells were activated for 24 h and the cell surface expression of CD83 **(A)** and the co-stimulatory molecule CD80 **(B)** was measured by flow cytometry. Mean fluorescence intensity (MFI) and the ratio of CD83 positive cells were calculated from three independent experiments + SD. Histogram overlays are shown for one independent experiment **(C)**. Production of inflammatory cytokines TNFα, IL-1β, IL-6, IL-12 and the regulatory cytokine IL-10 in the supernatants of moDCs was detected after 24 h by ELISA. Mean values of cytokine concentrations were calculated from 3 independent experiments + SD **(D)**. Freshly isolated PBLs were co-cultured with autologous moDCs for 4 days. The number of cytokine-producing PBLs in response to LPS as control or MUB-exposed human moDCs was measured by IFNγ ELISPOT assay. Mean values of spot numbers were calculated from 3 independent experiments ± SD **(E)**. Statistical differences were analyzed by Student’s *t*-test, with significance defined as ^∗^*P* < 0.05, ^∗∗^*P* < 0.01, ^∗∗∗^*P* < 0.001, and ^∗∗∗∗^*P* < 0.0001.

To further characterize the immunomodulatory potential of *L. reuteri* strains, native MUB was purified from *L. reuteri* ATCC 53608 spent culture medium, either associated with glycolipids, or separated from the glycolipid fraction when CHAPS was employed (Supplementary Figure S1). We investigated the inflammatory nature of purified, endotoxin-free MUB as compared to LPS, and also the glycolipid fraction in the course of the moDC-regulated immune response. Increasing concentrations of purified MUB induced CD83 expression on the moDC surface (**Figures 5A,C**). The cell surface expression of the CD80 co-stimulatory molecule was also enhanced independent of MUB concentration, although this effect did not reach statistical significance (**Figures 5B,C**). The level of moDC-secreted pro-inflammatory cytokines, including TNF-α, IL-1β, IL-6, IL-12 and the anti-inflammatory cytokine IL-10 was dependent on the concentration of MUB, except in the case of IL-6. When moDCs were cultured in the presence of 0.5 or 1 μg/ml MUB, the production of IL-10 cytokine, known to have regulatory potential, was found to be in the logarithmic range of 0.01–0.1 ng/ml, while the concentration of the pro-inflammatory cytokine IL-12 varied within the 0.1–1 ng/ml range (**Figure 5D**). Furthermore, moDC cultures activated with LPS or MUB induced Th1 polarized immune responses associated with increased IFNγ production (**Figure 5E**), in line with the increased concentration of IL-12. ATRA had no effect on the purified MUB-induced moDC response (data not shown). The extracted lipid fraction also induced CD83 and CD86 expression on the moDC cell surface to a similar extent as induced by the lipid containing MUB fraction (Supplementary Figure S2A). However, pure MUB lacking glycolipids was associated with the reduced secretion of IL-12 and was unable to induce IL-23 production by moDCs (Supplementary Figure S2B). Furthermore, the lipid fraction was unable to induce IL-12 or IL-23 production, suggesting that the immunogenicity of MUB can be enhanced by the lipid component of this fraction.

### The Immunomodulatory Properties of MUB Are Mediated by DC-SIGN and Dectin-2 Interactions in moDC

**FIGURE 6:**
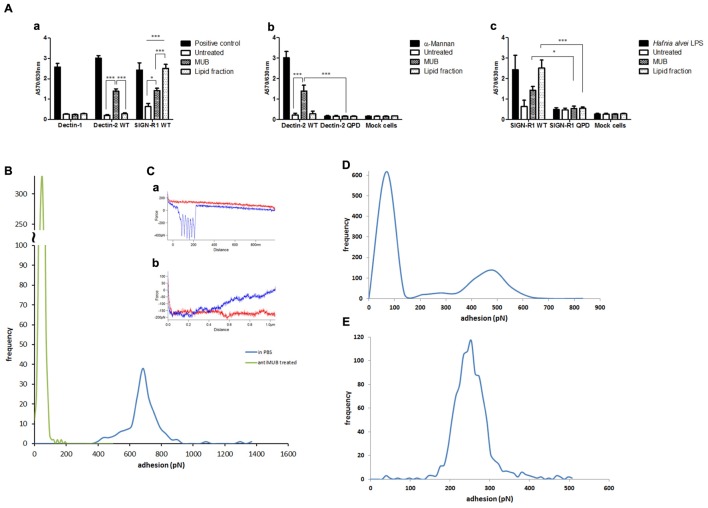
**Interaction of MUB with C-type lectin receptors. (A)** Cell reporter assays were carried out to screen the interaction of MUB and MUB-derived lipid with CLRs **(a)**, mDectin-2 and QPD-mutant reporter cells **(b)**, SIGN-R1 and QPD-mutant reporter cells **(c)**. Scleroglucan, α-mannan, and *Hafnia alvei* LPS were used as a positive control for mDectin-1, mDectin-2, and SIGN-R1, respectively. Statistical differences were determined by ANOVA, with significance defined as ^∗^*P* < 0.05, ^∗∗^*P* < 0.01, and ^∗∗∗^*P* < 0.001. Force spectroscopy was used to measure the direct interaction between MUB functionalized AFM tip and CLRs. **(B)** Adhesion histograms between MUB and DC-SIGN. **(C)** Example of interaction force spectroscopy curves (Red – approach, Blue – retract) **(a)** MUB tip against DC-SIGN in PBS **(b)** MUB tip against DC-SIGN in presence of anti-Mub antibody **(D)** Adhesion histogram of MUB and SIGNR1. **(E)** Adhesion histogram of MUB and Dectin-2.

To gain insight into the mechanisms mediating the interaction of MUB with moDCs, we first tested the binding of MUB to reporter cells expressing specific CLRs on the cell surface. MUB significantly bound to murine mDectin-2 and SIGN-R1 (murine analog of human DC-SIGN) reporter cells in a dose-dependent manner (**Figure 6A**). There was no binding to the mock cells (used as a negative control) and we could not detect any binding of MUB to mDectin-1 using the same approach. When MUB was incubated with reporter cells expressing the mDectin-2 QPD mutant, in which the mannose-binding activity was eliminated by substituting EPN (glutamic acid-proline-asparagine) sequence into galactose-type QPD, the binding was reduced, indicating the specificity of the interaction. Interestingly, the lipid fraction showed strong interaction with the SIGN-R1 receptor, at a level similar to that of the positive control, *Hafnia alvei* LPS, which has α-linked mannan *O*-antigen (Wittmann et al., 2016). The binding of MUB to CLRs was further investigated by atomic force spectroscopy. In this experiment, MUB was covalently linked to the functionalized AFM tip, and the CRLs were immobilized onto the glass slide, taking advantage of specific covalent attachment chemistry (Bhatia et al., 1989). The modal value of binding strength (684 pN) revealed a strong interaction between MUB and DC-SIGN (**Figure 6B**). The addition of anti-Mub antibody to the liquid cell significantly inhibited the interaction. The presence of highly symmetrical negative peaks in both force magnitude and distance indicates sequential unfolding of Mub multiple repeats as DC-SIGN and MUB are pulled apart (Carrion-Vazquez et al., 1999) (**Figure 6C**). The number of unfolding events was significantly reduced in the presence of anti-Mub antibody (**Figure 6C**). **Figures 6D,E** show the quantification of the binding interactions between MUB and SIGN-R1 or Dectin-2, respectively.

**FIGURE 7:**
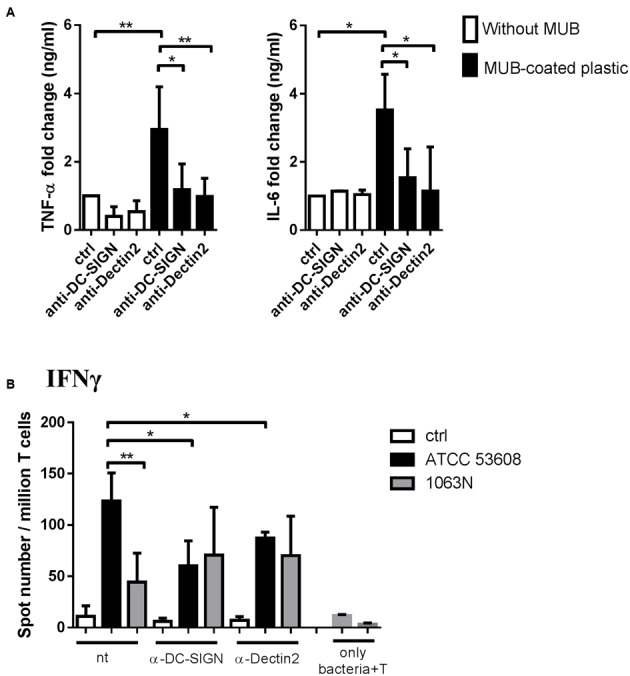
**The molecular background of bacterial MUB adhesin – moDC interaction causing inflammation and T-lymphocyte polarization. (A)** MUB immobilized on a microtiter plate was incubated with 2-day moDC cultures treated with anti-Dectin-2 or anti-DC-SIGN antibodies. Unlabeled (ctrl) moDCs incubated on MUB-coated and uncoated wells served as negative and positive controls, respectively. The production of pro-inflammatory TNF-α and IL-6 cytokines was measured by ELISA after 24 h. Mean value of cytokine concentrations was calculated from 5 independent experiments + SD. **(B)** MoDCs treated with anti-Dectin-2 or anti-DC-SIGN antibodies were co-cultured with live *L. reuteri* ATCC 53608 and 1063N strains for 1.5 h at 37°C, respectively. Freshly isolated PBLs were co-cultured with autologous moDCs for 4 days. The number of cytokine-producing T-cells was measured by IFNγ ELISPOT assay. Mean values of spot numbers were calculated from 5 independent experiments + SD. T corresponds to T-cells cultured without dendritic cells as negative control. Statistical differences were analyzed by Student’s *t*-test, with significance defined as ^∗^*P* < 0.05, ^∗∗^*P* < 0.01, ^∗∗∗^*P* < 0.001, and ^∗∗∗∗^*P* < 0.0001.

To gain further insights into the enhanced inflammatory response induced by bacterial MUB in moDCs, we analyzed the role of selected CLRs, Dectin-2 and DC-SIGN, upon MUB–moDC interaction. We found that immobilized MUB activated the secretion of pro-inflammatory cytokine TNF-α and IL-6 (**Figure 7A**) and this effect could be prevented in moDCs efficiently by neutralizing the signal transducer function of Dectin-2 and DC-SIGN receptors with blocking antibodies to DC-SIGN or Dectin-2, while IL-1β and IL-12 cytokine production was not detected (data not shown). Interestingly, we found that *L. reuteri* uptake was not eliminated in moDCs in the presence of anti-DC-SIGN or anti-Dectin-2 antibodies (data not shown). To confirm the involvement of Dectin-2 and DC-SIGN in the activation of moDC-mediated T-cell differentiation, blocking antibodies targeting these cell surface CLRs were used in the presence of *L. reuteri* ATCC 53608 and 1063N (**Figure 7B**). The Th1 polarizing capacity of moDCs induced by the wild-type strain was reduced in presence of anti-Dectin-2 or anti-DC-SIGN antibodies as compared to CLR-unblocked moDCs. As expected, the Th1 polarizing capacity of the mutant strain 1063N was not affected by blocking DC-SIGN and Dectin-2 on moDCs. These results clearly demonstrate that the interaction of Dectin-2 and DC-SIGN with MUB presented by *L. reuteri* ATCC 53608 is crucial to acquire Th1-cell differentiation upon bacterial stimulation of moDCs.

## Discussion

Probiotic lactobacilli, as part of the healthy microbiota, have been reported to regulate mammalian cytokine production and intestinal inflammation in various experimental model systems (van Baarlen et al., 2013). However, it is also clear that the immunomodulatory effects of probiotic strains such as *L. reuteri* are strain-dependent, exerting different DC activation patterns *in vitro*. For example, *L. reuteri* strains ATCC PTA 6475 and ATCC PTA 5289 were reported to suppress the production of TNF-α by LPS-activated monocytic cells (Lin et al., 2008; Jones and Versalovic, 2009), whereas the *L. reuteri* strains ATCC 55730 and CF48–3A were shown to have an immuno-stimulatory effect (Lin et al., 2008; Jones and Versalovic, 2009). A similar down-regulation of pro-inflammatory cytokines (e.g., TNF-α) by *L. reuteri* ATCC PTA 6475 was also observed with primary monocyte-derived macrophages from children with Crohn’s disease (Peña et al., 2005). Christensen et al. (2002) showed that the *L. reuteri* DSM 12246 strain was a poor inducer of IL-12, TNF-α, and IL-6 in bone marrow-derived murine DCs. Moreover, a more recent study showed that *L. reuteri* strains from human-associated clades differed in respect to their ability to modulate human cytokine production (TNF-α, monocyte chemoattractant protein-1 (MCP-1), IL-1β, IL-5, IL-7, IL-12, and IL-13) by stimulated myeloid cells (Spinler et al., 2014). Although not well understood, it is likely that the sum of bacterial cell surface-derived and soluble factors and/or exopolysaccharides (EPS) contribute to the development of different immune responses induced by *L. reuteri* strains. For example, soluble factors of *L. reuteri* CRL1098 were able to reduce TNF-α production by human PBMCs (Mechoud et al., 2012) and in mouse models (Griet et al., 2014). Furthermore, human moDCs generated in the presence of the soluble factors of *L. reuteri* DSM 17938 down-modulated LPS-induced IL-6, IL-10, and IL-23 secretion while the secretion of regulatory cytokine TGF-β remained unaffected (Haileselassie et al., 2016). EPS isolated from *L. reuteri* strain DSM 17938 and L26 Biocenol^TM^ was recently shown to exert up-regulation of the mRNA level of IL-1β, NF-κB, TNF-α, and IL-6 (Kšonžeková et al., 2016). One of the putative *L. reuteri* surface proteins appeared to be important for the stimulation of THP-1 cells and the activation of NF-κB in U937-3xkB-LUC cells by *L. reuteri* strains (Jensen et al., 2015). However, the specific mechanisms by which bacterial molecules could modulate cytokine expression in antigen-presenting cells remains to be identified.

Here we showed that host strain-specific adhesins contribute to the immunomodulatory effect of *L. reuteri* ATCC PTA 6475 and ATCC 53608 mediating (i) increased adherence and phagocytosis of *L. reuteri* strains by moDCs, (ii) enhanced CD83 expression, (iii) induced secretion of pro-inflammatory cytokines (TNF-α, IL-1β, IL-6) and that of the T-lymphocyte polarizing cytokines IL-12 and IL-23, and (iv) Th1 and Th17-polarized immune responses characterized by IFNγ and IL-17 production, respectively. Both wild-type ATCC PTA 6475 and ATCC 53608 showed higher internalization by moDCs and increased expression of moDC activation markers as compared to the mucus adhesin mutant strains. However, strain-specific differences were observed in terms of cytokine production; TNF-α was induced preferentially in moDCs activated by the CmbA-expressing strain ATCC PTA 6475, while the MUB-expressing ATCC 53608 strain induced IL-1β and IL-6 secretion. It is worth noting that *L. reuteri* ATCC PTA 6475 is generally considered as anti-inflammatory due to its reported ability to inhibit or down-regulate pro-inflammatory cytokines such as TNF-α, MCP-1, IL-1β, and IL-12 in stimulated myeloid cells (Lin et al., 2008), consistent with the known ability of *L. reuteri* strain ATCC PTA 6475 to suppress intestinal inflammation induced by LPS (Liu et al., 2010). Here we showed that, in absence of stimulatory signals mediated by LPS-induced PRRs, the immunogenicity of *L. reuteri* strains was increased by mucus-binding adhesins, and that the mutant strains showed a more tolerogenic immune response represented by decreased Th1 and Th17 immune responses, an effect which involves the CLR-induced signaling pathways in moDCs.

C-type lectin receptors represent one subset of PRRs expressed by a broad spectrum of cells (Meyer-Wentrup et al., 2005) and recognize a diverse range of endogenous and exogenous ligands including fungi, bacteria, parasites, and danger-associated molecular patterns driving both innate and adaptive immunity (Gijzen et al., 2006; Sukhithasri et al., 2013). The mechanism by which MUB exerts immunomodulatory effects was further investigated using a cell reporter assay specific for different CLRs showing that purified MUB from *L. reuteri* ATCC 53608 was recognized by mDectin-2 and SIGN-R1 but not by mDectin-1. Dectin-1 is the major receptor on macrophages for β-1,3-glucan, a polymer of glucose present in the fungal cell wall (Herre et al., 2004) whereas Dectin-2, expressed by macrophages and various DC subsets recognizes high-mannose ligands found in diverse microbes, including *Candida albicans* (Zhu et al., 2013), *Malassezia furfur* (Ishikawa et al., 2013), and *Schistosoma mansoni* (Ritter et al., 2010). DC-SIGN (or CD209) is expressed in sub-epithelial DC subsets including CD1c^+^ and inflammatory DCs, both having the potential to modulate PRR-mediated signaling pathways (Soilleux, 2003; Geijtenbeek and Gringhuis, 2009). SIGN-R1, or CD209b, is one of the eight mouse homologs of human DC-SIGN (Park et al., 2001; Kang et al., 2003; Powlesland et al., 2006). These CLRs bind a broad variety of pathogenic microbial organisms and their polysaccharides (Geijtenbeek and Gringhuis, 2016) such as HIV virus (Geijtenbeek et al., 2000), mycobacteria (Geijtenbeek et al., 2003), *C. albicans* (Cambi et al., 2003; te Riet et al., 2015), *Streptococcus pneumoniae* (Kang et al., 2004) or *Helicobacter pylori* (Bergman et al., 2004) and serve as a target of penetration and infection in the host’s cell while the specific immune response is prevented (van Kooyk et al., 2003). The direct interaction of MUB with DC-SIGN, SIGN-R1 and Dectin-2 was confirmed by atomic force spectroscopy. We clearly demonstrated that purified MUB, either in solution or immobilized, induced the secretion of inflammatory cytokines in moDCs. Furthermore, secretion of TNF-α and IL-6, but not IL-1β or IL-12, was reduced by the neutralization of the Dectin-2 and DC-SIGN-mediated inflammatory signaling pathways. These results suggest that the enhanced level of inflammatory Th1 and Th17 immune responses provoked by wild-type *L. reuteri* ATCC 53608 is dependent on the intimate interaction between MUB and the surface C-type lectin receptors of moDCs. Surprisingly, the uptake of *L. reuteri* ATCC 53608 was not affected in moDCs upon the selective blocking of DC-SIGN or Dectin-2. On the other hand, DC-SIGN and Dectin-2 receptors played a crucial role in moDC-mediated Th1 type immune responses against *L. reuteri* ATCC 53608.

DC-SIGN present on immature DCs appears to be a general receptor for microbes leading to downstream T cell activation (Gijzen et al., 2007). DC-SIGN signaling stimulates tolerance responses in DCs, which is an important component in maintenance of homeostasis (Geijtenbeek and Gringhuis, 2009; Rabinovich, 2010; Wells, 2011) and can be elicited by specific lactobacilli (Penders et al., 2010) including strains of *L. rhamnosus* (Konieczna et al., 2015), *L. acidophilus* (Martínez et al., 2012), *L. reuteri* and *L. casei* (Smits et al., 2005). Interestingly, *Lactobacillus* cell surface proteins, such as *L. rhamnosus* proteinaceous pili (Tytgat et al., 2016) and *L. acidophilus* S-layer proteins (Konstantinov et al., 2008; Martínez et al., 2012; Prado Acosta et al., 2016) have recently been implicated in the interaction of *Lactobacillus* spp. with DC-SIGN. *L. rhamnosus* GG pili bind to mucus (Kankainen et al., 2009) and interact with macrophages (Vargas García et al., 2015). Recently, purified SpaCBA pili of *L. rhamnosus* GG were shown to directly interact with DC-SIGN in a carbohydrate-dependent manner and induce IL-6, IL-10, IL-12p40, and IL-12p35 expression in DCs (Tytgat et al., 2016). The induction of these cytokines was partially dependent on DC-SIGN (Tytgat et al., 2016), in agreement with the reported impact of pili on TLR-2 signaling (Douillard et al., 2013; von Ossowski et al., 2013), which might be modulated by DC-SIGN, and the ability of *L. rhamnosus* GG wild-type and mutant strains to modulate several pro- and anti-inflammatory cytokines (Lebeer et al., 2012; von Ossowski et al., 2013; Vargas García et al., 2015). Our results indicated that TNF-α and IL-6 secretion but no other inflammatory cytokines and chemokines was dependent on both Dectin-2 and DC-SIGN upon interaction with MUB, which suggest that other PRRs are also involved in this process. As far as we know, Dectin-2 has not been implicated in *Lactobacillus* immunomodulation (van Baarlen et al., 2013). Previous studies on fungal pathogens showed that the activation of Dectin-2 but not Dectin-1, led to the secretion of IL-23 and IL-1β, supporting the polarization of Th17 cells (Gringhuis et al., 2011; Loures et al., 2015). Here, *L. reuteri* 1063N mutant was less effective at inducing IL-1β secretion but no difference could be detected between the IL-23 secretion levels induced by the wild-type and the mutant *L. reuteri* strains. However, the prevention of molecular interaction between Dectin-2 and immobilized MUB reduced the production of IL-6, but not IL-1β, in moDCs indicating that IL-6 may serve as a pivotal factor in *L. reuteri* ATCC 53608-induced Th17 polarization.

Taken together, these data provide novel insights into the mechanisms by which *L. reuteri* strains exert immunomodulatory properties *via* the direct interaction of *L. reuteri* host-specific adhesins with C-type lectins on DCs. The mucus-binding adhesins expressed on the surface of *L. reuteri* bacteria may contribute to the maintenance of the symbiotic relationship with the host by acting as a natural adjuvant, thus provoking antigen-specific adaptive immune responses by moDCs through the development of effector and memory T-lymphocytes with sufficient stimulatory potential. Further research is warranted to assess the pivotal role of protein–glycan interactions in the immunomodulatory capacities of probiotic strains.

## Author Contributions

KB carried out all moDc assays. DK purified MUB and glycolipid. CL carried out all CLR cell reporter assays. AW and IY established the reporter cells for Dectin-1 and SIGN-R1, IY performed the SIGN-R1 cell reporter assay with Hafnia-LPS. AG performed the AFM force spectroscopy assays. DM carried out the *Lactobacillus reuteri* growth cultures. NK supervised the CLR cell reporter assays. ER supervised the moDC assays. NJ coordinated the work and writing up of the manuscript

## Conflict of Interest Statement

The authors declare that the research was conducted in the absence of any commercial or financial relationships that could be construed as a potential conflict of interest. The reviewer ADAF and handling Editor declared their shared affiliation, and the handling Editor states that the process nevertheless met the standards of a fair and objective review.
