# Supplementary material for: Lactobacillus reuteri Surface Mucus Adhesins Upregulate Inflammatory Responses Through Interactions With Innate C-Type Lectin Receptors
Source: Front Microbiol. 2017 Mar 7;8:321. doi: 10.3389/fmicb.2017.00321 (PMC5339304; doi:10.3389/fmicb.2017.00321)
Supplement: Supplementary file 1 [file Data_Sheet_1.DOCX]

**Supplementary material**

**Fig. S1 MUB and glycolipid separation**. SDS-PAGE gel (4-12%) of the eluted fractions collected during the purification of MUB. Early, high molecular weight fractions are MUB protein (left), with the MUB-associated glycolipid eluting in later fractions, precipitating in-gel, and identified by reflective white light (right).

**
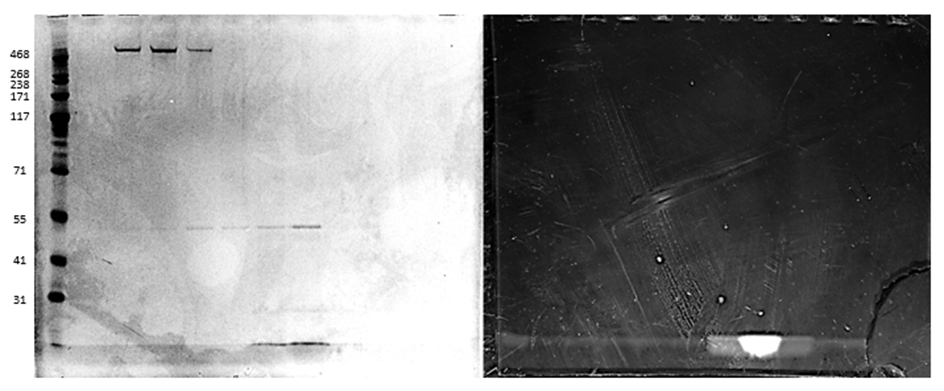
**

**Fig. S2 Immunomodulatory properties of MUB-associated glycolipids.**

Purified MUB protein, lipid containing MUB and lipid extract were tested for their moDC- activating capacity in a 2-day moDC culture. LPS served as a positive control, the spent culture media, PBS, distilled water and CHAPS were used as negative controls. Cells were activated for 24 h and the cell surface expression of CD83 and co-stimulatory molecule CD80 was measured by flow cytometry (**A**). Mean fluorescence intensity (MFI) and the ratio of CD83 positive cells were calculated from 3 independent experiments +SD. Production of the T-cell polarizing cytokines IL-12 and IL-23 was detected after 24 h from the supernatants of moDCs by ELISA (**B**). Mean values of cytokine concentrations were calculated from 3 independent experiments + SD.

**CD83**

**A**

**B**

**CD86**

**IL-12**

**IL-23**
